# Prevalence of All-Cause Mortality and Suicide among Bariatric Surgery Cohorts: A Meta-Analysis

**DOI:** 10.3390/ijerph15071519

**Published:** 2018-07-18

**Authors:** Russell B.C. Lim, Melvyn W.B. Zhang, Roger C.M. Ho

**Affiliations:** 1 Department of Psychological Medicine, Yong Loo Lin School of Medicine, National University of Singapore, Singapore 119074, Singapore; lim.boonchai.russells@gmail.com (R.B.C.L.); pcmrhcm@nus.edu.sg (R.C.M.H.); 2 National Addictions Management Service, Institute of Mental Health, Singapore 539747, Singapore

**Keywords:** mortality, suicide, bariatric surgery, meta-analysis

## Abstract

Introduction: Prior meta-analysis has reported mortality rates among post-operative bariatric patients, but they have not considered psychiatric factors like suicide contributing to mortality. Objectives: The current meta-analysis aims to determine the pooled prevalence for mortality and suicide amongst cohorts using reported suicides post bariatric surgery. It is also the aim of the current meta-analytical study to determine moderators that could account for the heterogeneity found. Results: In our study, the pooled prevalence of mortality in the studies which reported suicidal mortality was 1.8% and the prevalence of suicide was 0.3%. Mean body mass index (BMI) and the duration of follow-up appear to be significant moderators. Conclusions: Given the prevalence of suicide post bariatric surgery, it is highly important for bariatric teams to consider both the medical and psychiatric well-being of individuals pre- and post-operatively.

## 1. Introduction

In 2014, the World Health Organization reported that there are an estimated 1.9 billion adults who were overweight, with approximately 600 million deemed to be obese (with a body mass index of more than 30) [1]. These figures highlight that obesity is clearly a problem that afflicts a global population. The increasing rates of obesity globally can be attributed mainly to changing dietary norms, as well as decreasing levels of physical activity among individuals [1]. Obesity heightens the risk of an individual developing medical comorbidities, such as diabetes, cardiovascular diseases and some forms of carcinomas [2]. Apart from the association between obesity and medical disorders, prior research has highlighted an association between obesity and psychiatric disorders [3]. In the aforementioned review [3], there was evidence of a bi-directional association of obesity and depressive disorders and females, in particular, tend to be at risk. An association between obesity and anxiety disorders was also found, and alcohol abuse appears to be a predisposing factor towards the development of obesity [3].

Various strategies have been implemented in order to tackle the growing problem of obesity. Such strategies include educational and dietary programs [4]. While these programs are largely efficacious, there are individuals who remains severely obese, despite their participation in these programs. Various clinical guidelines recommend bariatric surgery as an option for individuals who have recalcitrant obesity, that is, refractory to non-operative management [5,6]. Bariatric surgery has been widely utilized, with approximately 468,809 procedures done in 2013 [7], and has been effective in the management of obesity. There is a variety of operative procedures that can be performed, and the amount of weight loss is dependent on the procedure utilized. Aside from the main benefits of weight loss, individuals who have undergone bariatric surgery may also gain optimal control of their diabetic and hypertensive disorders and resolution of obstructive sleep apnoea [8,9,10].

While bariatric surgery seems to be a solution to obesity as well as the associated medical comorbidities, prior studies have highlighted that there remains an elevated risk of suicide among patients who have undergone bariatric surgery [11]. A prior meta-analysis reported that the suicide rate was 4.1/10,000 person-years among post-operative bariatric patients, and this was four times higher as compared to the general population [12]. Other studies (Tindle et al.) [13] have reported rates of suicide as high as 6.6/10,000 patients. Obesity is associated with a variety of psychiatric and affective disorders, for example, depressive disorders are commonly associated with obesity. A recent study of 10,000 bariatric patients in Canada reported that 41.7% of patients had depression and 2.2% had bipolar disorder pre-operatively [14]. There have been studies reporting that the risk of post-operative suicide remains high [15]. A prior study reported that self-esteem might account for the relationship between obesity and suicidality [16]. Self-esteem affect one’s viewpoint of one’s body image and individuals with low self-esteem might have a poorer perception of their body image, which leads to the development of depression [16]. In addition, some of the chronic medical comorbidities may persist despite bariatric surgery and the presence of these medical issues might cause an individual to have a sense of failure and disappointment [17]. It is also important to recognize that the risk of suicide is increased among individuals with disorders such as diabetes. Bariatric surgery can also result in physiological changes, and this may affect the metabolism of substances like alcohol, which might be implicated in suicide attempts. Changes in the levels of peripheral released peptides might also affect mood. Patients with pre-existing emotional eating disorders might still have maladaptive patterns of eating post-operatively that lead to weight gain and result in a sense of disappointment. 

Previous studies have reported short-term as well as long-term all-cause mortality (Cardoso et al., 2017) [11]. The estimated prevalence of short-term mortality was 0.18% and it was also reported that operated patients were less likely to succumb to cardiovascular disorders and carcinomas in the longer-term. Whilst the meta-analysis performed by Cardoso et al. (2017) [11] appears to be comprehensive and timely, one of the major limitations is that the authors have not considered mortality due to suicide in their review. Also, the prior study [12] examining the prevalence of completed suicide was done more than 4 years ago. Hence, a current meta-analysis is of importance in providing an updated pooled prevalence rate for suicide, and for comparing it against that of all-cause mortality in the same cohort. 

Thus, this meta-analysis aims to determine the pooled prevalence of mortality and suicide among the cohort of bariatric surgery patients with reported suicides. It is also the aim of the meta-analysis to determine the moderators that account for the heterogeneity of the pooled prevalence obtained. 

## 2. Methodology

### 2.1. Comprehensive Search Strategy

A comprehensive search was undertaken between 1 January 2017 and 28 February 2017. Entire databases were searched from inception with the following databases being evaluated: PubMed (since 1966), Embase (since 1980), PsychINFO (since 1806), BIOSIS (since 1926), Science Direct (since 2006), and Cochrane CENTRAL (since 1993). 

The keywords used in the search strategy include; (obesity surgery, bariatric surgery, gastric bypass, biliopancreatic diversion, endoluminal sleeve, vertical banded gastroplasty, gastric band, sleeve gastrectomy, gastric balloon, gastric plication, duodenal switch, implantable gastric stimulation) AND (suicide OR mortality). 

### 2.2. Inclusion and Exclusion Criteria

The inclusion criteria for the meta-analysis were as follows: (1) papers which provided numbers of suicide in the cohort, and (2) papers with recipients of bariatric surgery form the study population.

The exclusion criteria for the meta-analysis were as follows: (1) non-English language papers, and (2) papers with unclear/unknown causes of death listed.

### 2.3. Selection of Articles 

All the titles, authors’ information, as well as the journal and year of publication were removed prior to the selection procedure. Selection of the relevant publications were conducted independently by of the first two authors (RBCL & MWBZ) of this paper. In the first phase, articles were screened based on their titles as well as abstract. Those articles which were shortlisted were then evaluated against the aforementioned inclusion and exclusion criteria. In the event of any disagreement amongst the two authors, it was resolved by means of a discussion with the author, RCMH. The selection procedure was in accordance to PRISMA guidelines.

### 2.4. Statistical Methods

#### 2.4.1. Data Extraction

The following information was extracted from each of the article, cross-checked by the second author as well as the last author and recorded on a standardized electronic data collation form: (a) publication details (names of the authors and year of publication); (b) the total number of deaths as well as the number of suicides; (c) the total sample size of each of the studies; (d) the mean age of the participants; (e) the proportion of males and females in the population surveyed; (f) the mean BMI of the participants; (g) the operative procedure utilized; (h) the country in which the participant were sampled from, and lastly, (i) the total duration of the follow-up. 

#### 2.4.2. Statistical Analysis

All statistical analyses were performed using comprehensive meta-analysis. This meta-analysis used a random-effects model that assumed heterogeneity between studies and their respective effect sizes (Ho et al. 2010, Cheung et al. 2012) [18,19]. We used standardized mean difference to establish the overall effect size in each of the studies and presented our findings in the forest plots. We reported the results using 95% confidence interval (CI). Between-study heterogeneity was assessed with the I^2^ statistic (Loh et al. 2017) [20]. As a guide, I^2^ values of 25% were considered low, 50% moderate, and 75% high (Ho et al. 2016) [21]. For models with considerable heterogeneity, a meta-regression was performed to identify the moderators which might contribute to the heterogeneity of the effect sizes (Lu et al. 2012) [22]. The regression coefficients and the associated z values and p values were reported in the meta-regression analysis. In the event that publication bias was detected, the classic fail-safe test was performed to establish the potential number of missing studies (Puthran et al. 2016) [23]. Egger’s regression test was also conducted to determine if publication bias was present. 

Two separate subgroup analyses were undertaken to investigate the effects of categorical variables on the pooled prevalence of mortality amongst cohort with reported suicide and on the pooled prevalence of suicide itself. We compared the prevalence of suicide among the following subgroups: (a) operative procedure that was utilized and (b) continent in which the study was conducted.

## 3. Results

**Figure 1:**
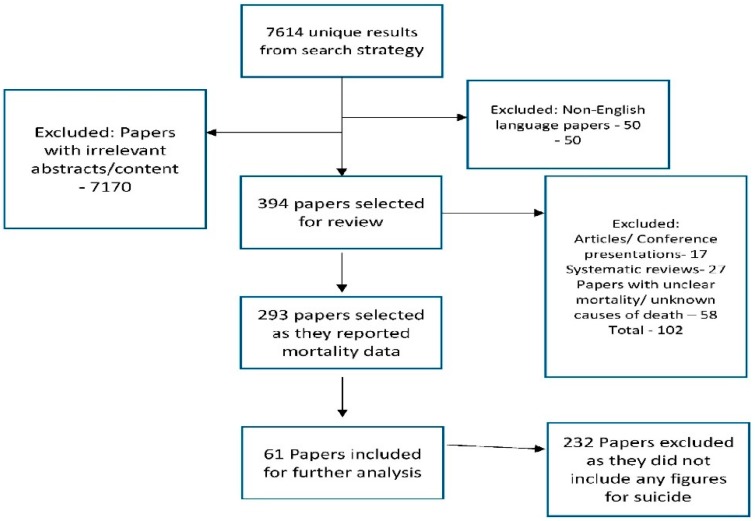
Flow chart showing the selection of the studies.

A cumulative total of 7614 published abstract were screened and 390 full text articles were reviewed and were selected based on our inclusion criteria. Sixty-one studies with a pooled cohort size of 142,356 were included in this systematic review and meta-analysis (Figure 1). Characteristics of the studies included are described in Table 1. There was a total of 43 prospective cohort studies, 14 retrospective cohort studies, one randomized controlled study and three case control studies. The studies which we identified have reported all-cause mortality and mortality due to suicide. 

Overall, the pooled prevalence of mortality in these studies which reported suicidal mortality was 1.8% (95% confidence interval 1.4–2.4%, Z = −29.228, df = 60, τ^2^ = 0.933, I^2^ = 95.779). This meta-analysis revealed significant heterogeneity across studies (*p* < 0.001). The pooled prevalence of suicide was 0.3% (95% confidence interval 0.3–0.4%, Z = −39.133, df = 60, τ^2^ = 0.684, I^2^ = 66.202). 

We also tested for publication bias using the Egger regression test. Publication bias was not evident in the meta-analysis of all the studies (intercept = −0.98486, 95% CI: −2.79555–0.82584, t = 1.08836, df = 59, *p* = 0.28086).

In the meta-regression analyses (Table 2), certain variables were found to be significantly associated with the overall pooled mortality prevalence. We found that the mean BMI (β = 0.008282, Z = 2.37980, *p* = 0.01732) and the follow-up interval (β = 0.01177, Z = 4.34545, *p* = 0.00001) were significant moderators for the pooled mortality prevalence. The mean age of the sampled cohort as well as the proportion of males in the sampled cohort were not found to be moderators.

Subgroup analysis of the prevalence rates based on the random effects model for the categorical variables (the types of bariatric surgical procedure as well as the continent where the cohort was sampled) found that these variables were not moderators for the overall prevalence of mortality among bariatric cohorts with reported suicide mortality (Table 3).

A further subgroup analysis was performed for the prevalence rates of suicide based on the random effects model for categorical variables (the types of bariatric surgical procedure as well as the continent where the cohort was sampled) and found that these variables were not moderators for the overall prevalence of mortality among bariatric patients (Table 4).

## 4. Discussion

The current meta-analysis is, to our knowledge, the most up-to-date meta-analysis to examine the pooled prevalence of all-cause mortality as well as suicide in bariatric surgery cohorts with reported suicides. In our current study, the pooled prevalence of all-cause mortality was 1.8% across a total of 61 studies with a pooled cohort size of 142,356. The pooled prevalence of suicide was 0.3%. Notably, our computed pooled prevalence rates were much higher than those reported by prior studies, such as that of Cardoso et al. (2017) [11], in which it was reported that the short-term all-cause mortality rate was 0.18%; and that of Chang et al. [84], that reported a mortality rate of 0.08%. Based on our computation, the rate of all-cause mortality is approximately 6 times higher than that for suicide. This implies that some bariatric patients do experience other complications that might result in morbidity and eventual mortality. Rottensterich et al. (2016) [85] has reported that whilst bariatric surgery helps in the weight loss amongst individuals with Type 1 diabetes, some individuals experience post-operative complications such as diabetic ketoacidosis and hypoglycaemic episodes. Prior studies have reported that factors such as gender, age, high baseline body mass index, the presence of pre-existing diabetes, history of percutaneous coronary intervention, a history of peripheral vascular disease and a need for reoperation heighten the chances of post-operative mortality [86].

The pooled prevalence of suicide after bariatric surgery was 0.3%. This is a notable finding, given that most of the recent meta-analysis and systematic reviews have not reported on mortality due to psychiatric conditions. The most recent review that has considered suicide following bariatric surgery was conducted by Peterhansel et al. (2012) [12], who reported that the suicide rate was estimated to be that of 4.1/10,000 person-years. To put these rates into perspective, the prevalence of suicide globally is 1.4%, based on an epidemiological study by the World Health Organization [87]. Despite the fact that the pooled prevalence of suicide is 6 times lower than that of all-cause mortality, and that the rates are also comparatively lower as compared to the rates among the general population (1.4%), there is still a need for a comprehensive evaluation of the psychiatric well-being of individuals both pre and post-operatively. Prior studies have highlighted the association between bariatric surgery and suicide (Adam et al., 2015) [88]. Roziblatt et al. (2016) [89] suggested that individuals who have pre-existing psychiatric conditions such as depression and eating disorders, are more likely to be at risk for suicide post-surgery. Thus, given this heightened risk, Roziblatt et al. (2016) [89] recommended the need for a psychiatrist to follow up with the patient prior to and after their slated surgery. Yen et al. (2016) [15] reported that 40% of bariatric patients have underlying psychiatric disorders and stressed that it is of importance for early identification and optimization of these conditions, as they in turn affect the outcome of the surgery. Yen et al. (2016) [15] also recommended various non-pharmacological options such as psychotherapy to help individuals with their depressive symptoms post-surgery. Based on the characteristics of the study presented in Table 1, there has been a general increase in the number of suicides following bariatric surgery, with clusters of cases being more frequently reported in 2007 and 2010, and especially so in 2010, where a single study (Tindle et al., 2010) [13] reported a total of 31 suicides. More recently, in 2015–2017, there was an increase in the number of suicides as well, with a single study reporting 17 suicides (Laggeros et al., 2017) [46]. Given the risk of suicide associated with bariatric surgery and the incidence of psychiatric disorders among individuals undergoing bariatric surgery, it is important to have a multi-disciplinary team caring for these individuals. Based on the best practice guidelines, it is of importance to have a psychiatrist, psychologist and social worker as part of the psychosocial care team [90] and it is also essential for these healthcare professionals to have prior experience with working with such patients. Some of the commonly used questionnaires used for psychiatric assessment include the Beck-Depression Inventory, the Symptom Checklist-90-Revised (SCL-90-R), the Eating Disorder Inventory-2, Beck Anxiety Inventory and the Eating Disorders Examination (EDE-Q). Psychological interventions, in particular cognitive behavioral therapy, have been most widely used in the treatment, and patients routinely attend up to twelve sessions. 

Our current study also identified the mean body mass index (BMI) as well as the duration of follow-up to be significant moderators of the heterogeneity found in the pooled prevalence for mortality. The fact that body mass index (BMI) mediates the heterogeneity of the pooled prevalence is not surprising given that a previous study by Padwal et al. (2013) [91] tried to determine the importance of BMI as a mortality predictor. Padwal et al. (2013) [91] found that BMI did have an effect on the absolute rate of mortality and in their study, the odds ratio computed was 1.03. In the current meta-analytic study, the duration of follow-up was found to be a significant moderator and we hypothesize that a longer-term follow-up would affect the mortality measures, given that there are short-term and longer-term causes that could lead to mortality (Cardoso et al., 2017) [11].

There are several strengths of this current review. We comprehensively searched through the literature and looked at all studies that have reported mortality as well as suicide, and we have included studies that reported both statistics, in order to compare the overall pooled prevalence rates. Meta-analytical regression analysis as well as subgroup analysis were performed. However, the current study has several inherent limitations. These include 50 non-English language papers and 58 studies with unclear or unknown causes of mortality listed that were excluded for ease of analysis. In addition, suicide data amongst the analysed papers is also sparse, with data about the demographics, reasons, and time from surgery being generally available. Also, most studies failed to report how they managed to obtain information about deaths (such as whether they screened death records, etc.). Additionally, deaths from alcoholic cirrhosis, drug overdoses, poisonings, and accidents not explicitly stated as suicide were excluded from analysis. Most papers analysed also listed low rates of long-term follow-up. Subgroup analysis was performed using the longest follow-up period recorded as most studies did not state clear default rates and average follow-up duration for extraction. It is possible that suicide and mortality occur amongst subjects lost to follow-up, which may further increase the pooled prevalence of suicide and mortality as compared to the given results.

## 5. Conclusions

The current study computed the pooled prevalence of all-cause mortality as well as that of suicide among cohorts with reported suicide following bariatric surgery. The findings from the current meta-analysis have resultant clinical implications. There is a need for a multi-disciplinary team to look into the psychological well-being of bariatric patients pre and postoperatively.

## Figures and Tables

**Table 1:** Characteristics of the included studies.

	Author and Year	Study Type	Sample Size (n)	Mortality (n)	Suicide (n)	Mean Age	% Male	% Female	Mean BMI	Procedure	Country	Longest Follow-Up (Month)
1	Aarts et al. 2014 [24]	Prospective Cohort Study	201	6	1	37	0.23	0.77	45.6	Laparoscopic adjustable gastric banding	The Netherlands	216
2	Adams et al. 2007 [25]	Retrospective Cohort Study	9949	288	21	39.3	0.14	0.86	44.9	Roux- En-Y Gastric Bypass	USA	216
3	Adams et al. 2012 [26]	Prospective Cohort Study	1156	12	4	42.5	0.18	0.82	45.9	Roux- En-Y Gastric Bypass	USA	72
4	Arapis et al. 2017 [27]	Prospective Cohort Study	897	6	1	39.5	0.13	0.87	39.5	Laproscopic adjustable gastric banding	France	228
5	Arman et al. 2016 [28]	Prospective Cohort Study	106	2	1	38.5	0.26	0.74	38.5	Laproscopic Sleeve Gastrectomy	Belgium	140
6	Biertho et al. 2010 [29]	Retrospective Cohort Study	810	25	5	41.1	0.21	0.79	44.2	Biliopancreatic diversion with duodenal switch	Canada	201
7	Bolckmans et al. 2016 [30]	Retrospective Cohort Study	153	9	1	40.6	0.16	0.84	46.4	Biliopancreatic diversion with duodenal switch	Belgium	135
8	Busetto et al. 2007 [31]	Case control study	821	8	1	38.2	0.24	0.76	48.6	Laproscopic adjustable gastric banding	Italy	120
9	Busetto et al. 2014 [32]	Prospective Cohort Study	318	15	1	38.6	0.18	0.82	46.7	Laproscopic adjustable gastric banding	Italy	120
10	Cadiere et al. 2011 [33]	Retrospective Cohort Study	470	1	1	40	0.166	0.834	NA	Laproscopic gastric bypass	Belgium	66
11	Capella et al. 1996 [34]	Prospective Cohort Study	888	8	3	37	0.178	0.822	52	Vertical banded gastroplasty/Vertical banded gastroplasty-Roux-en-y gastric bypass	USA	60
12	Carelli et al. 2010 [35]	Retrospective Cohort Study	2909	10	1	NA	0.32	0.68	45.27	Laproscopic adjustable gastric banding	USA	60
13	Christou et al. 2006 [36]	Retrospective Cohort Study	272	8	2	42	0.18	0.82	48.1	Roux- En-Y Gastric Bypass	Canada	NA
14	Cobourn et al. 2013 [37]	Retrospective Cohort Study	2815	9	1	43	0.18	0.82	44.6	Laproscopic Adjustable Gastric Banding	Canada	60
15	Diniz et al. 2013 [38]	Prospective Cohort Study	248	9	2	39.7	0.25	0.75	53	Roux- En-Y Gastric Bypass	Brazil	NA
16	Goldfeder et al. 2006 [39]	Retrospective Cohort Study	107	107	1	NA	NA	NA	NA	NA	USA	NA
17	Girbsholt et al. 2016 [40]	Retrospective Cohort Study	9895	91	10	40.2	0.217	0.783	46	Roux- En-Y Gastric Bypass	Denmark	50
18	Higa et al. 2000 [41]	Prospective Cohort Study	1040	5	1	NA	0.174	0.826	47.8	Roux- En-Y Gastric Bypass	USA	12
19	Himpens et al. 2011 [42]	Prospective Cohort Study	82	3	1	50	0.1	0.9	41.57	Laproscopic Adjustable Gastric Banding	Belgium	NA
20	Himpens et al. 2012 [43]	Prospective Cohort Study	77	2	1	38.9	0.2	0.8	40.3	Laproscopic Roux-En-Y Gastric bypass	Belgium	112
21	Kelles et al. 2014 [44]	Prospective Cohort Study	4344	82	8	34.9	0.21	0.79	42	Roux- En-Y Gastric Bypass	Brazil	120
22	Kral et al. 1993 [45]	Prospective Cohort Study	69	3	1	38	0.18	0.82	47	Vertical Banded Gastroplasty	Sweden	60
23	Laggeros et al. 2017 [46]	Retrospective Cohort Study	22,539	NA	17	41.3	0.753	0.247	NA	NA	Sweden	26
24	Lemanu et al. 2015 [47]	Prospective Cohort Study	96	3	1	46.9	0.182	0.818	50.7	Laproscopic Sleeve Gastrectomy	New Zealand	60
25	Macdonald et al. 1997 [48]	Prospective Cohort Study	154	14	1	41.9	0.234	0.766	50.6	Roux- En-Y Gastric Bypass	USA	132
26	Maclean et al. 2000 [49]	Prospective Cohort Study	274	3	1	NA	NA	NA	43.2	Isolated Gastric Bypass	Canada	NA
27	Marceau et al. 2007 [50]	Prospective Cohort Study	1423	67	6	40.1	NA	NA	51.5	Duodenal Switch	Canada	180
28	Marceau et al. 2009 [51]	Prospective Cohort Study	686	49	3	NA	NA	NA	48.3	Biliopancreatic diversion with distal gastrectomy/Biliopancreatic diversion with duodenal switch	Canada	120
29	Marceau et al. 2015 [52]	Prospective Cohort Study	2615	123	9	42	0.307	0.693	52	Biliopancreatic diversion	Canada	NA
30	Marsk et al. 2010 [53]	Case control study	1216	43	4	NA	1	0	NA	NA	Sweden	NA
31	Mcphee et al. 2015 [54]	Case control study	206	2	2	NA	NA	NA	NA	Laproscopic sleeve gastrectomy/Laproscopic adjustable gastric banding	USA	NA
32	Mitchell et al. 2001 [55]	Prospective Cohort Study	78	8	1	56.8	0.17	0.83	43.8	Gastric bypass	USA	180
33	Naslund et al. 1994 [56]	Prospective Cohort Study	37	5	2	37	0.18	0.82	45.5	Gastric banding	Sweden	120
34	Naslund et al. 1995 [57]	Prospective Cohort Study	158	3	1	39.3	0.16	0.84	44.7	Vertical Banded Gastroplasty	Sweden	NA
35	Nocca et al. 2007 [58]	Prospective Cohort Study	163	1	1	41	0.32	0.68	45.9	Laproscopic Sleeve Gastrectomy	France	24
36	N Obeid et al. 2015 [59]	Prospective Cohort Study	328	9	2	41.4	0.17	0.83	47.5	Roux- En-Y Gastric Bypass	USA	120
37	O’Brien et al. 2013 [60]	Prospective Cohort Study	3227	4	1	47.1	0.22	0.78	43.8	Laproscopic Adjustable Gastric Banding	Australia	120
38	Omalu et al. 2007 [61]	Retrospective Cohort Study	16,683	440	16	48	0.177	0.823	NA	NA	USA	NA
39	Peeters et al. 2007 [62]	Cohort study	966	4	1	47	0.23	0.77	44.9	Laproscopic Adjustable Gastric Banding	Australia	NA
40	Pekkarinen et al. 1994 [63]	Prospective Cohort Study	33	3	1	36	0.33	0.67	50	Vertical Banded Gastroplasty	Finland	NA
41	Pories et al. 1992 [64]	Prospective Cohort Study	515	23	3	NA	0.15	0.85	NA	Greenville Gastric Bypass	USA	132
42	Pories et al. 1995 [65]	Prospective Cohort Study	608	34	3	37.3	0.168	0.832	NA	Greenville Gastric Bypass	USA	168
43	Powers et al. 1992 [66]	Prospective Cohort Study	100	2	1	38.8	0.15	0.85	47	Vertical Banded Gastroplasty	USA	NA
44	Powers et al. 1007 [67]	Prospective Cohort Study	131	5	1	39.4	0.15	0.85	NA	NA	USA	NA
45	Rawlins et al. 2012 [68]	Prospective Cohort Study	55	2	1	44	0.3	0.7	65	Laproscopic Sleeve Gastrectomy	USA	60
46	Rutte et al. 2014 [69]	Prospective Cohort Study	1041	6	1	42.5	0.29	0.71	44.3	Sleeve gastrectomy	The Netherlands	60
47	Shah et al. 2016 [70]	Prospective Cohort Study	3795	14	1	42.4	0.21	0.79	40.9	Roux- En-Y Gastric Bypass	Norway	60
48	Sieber et al. 2013 [71]	Prospective Cohort Study	68	1	1	43.1	0.22	0.78	43	Laproscopic Sleeve Gastrectomy	Switzerland	NA
49	Skroubis et al. 2010 [72]	Prospective Cohort Study	1162	21	2	36.3	0.26	0.74	53	Vertical Banded Gastroplasty/Laproscopic sleeve gastrectomy/Roux-en-y gastric bypass/Biliopancreatic diversion	Greece	NA
50	Smith et al. 1995 [73]	Retrospective Cohort Study	3855	24	2	NA	0.11	0.89	NA	Roux- En-Y Gastric Bypass	USA	84
51	Smith et al. 2004 [74]	Retrospective Cohort Study	779	2	1	39.3	NA	NA	40.32	Roux- En-Y Gastric Bypass	USA	29
52	Suter et al. 2006 [75]	Prospective Cohort Study	317	5	1	38	0.136	0.864	43.5	Laproscopic Adjustable Gastric Banding	Switzerland	84
53	Suter et al. 2011 [76]	Prospective Cohort Study	379	9	2	39.4	0.26	0.74	46.3	Roux- En-Y Gastric Bypass	Switzerland	84
54	Svenheden et al. 1997 [77]	Prospective Cohort Study	95	2	1	NA	0.21	0.79	42.5	Vertical Banded Gastroplasty	Sweden	24
55	Tao et al. 2014 [78]	Retrospective Cohort Study	22,487	85	1	NA	NA	NA	NA	Gastric bypass/Gastric Banding/Vertical Banded Gastroplasty/Laproscopic Sleeve Gastrectomy/Billiopancreatic Diversion with duodenal Switch/Jejunoileal bypass	Sweden	12
56	Thereaux et al. 2014 [79]	Prospective Cohort Study	330	7	1	43.4	0.089	0.911	46.9	Laproscopic Roux-En-Y Gastric bypass	France	60
57	Tindle et al. 2010 [13]	Prospective Cohort Study	16,683	NA	31	48	0.177	0.823	NA	Various	USA	NA
58	Van de Weijgert et al. 1999 [80]	Prospective Cohort Study	153	10	1	34	0.131	0.869	46	Roux-en-Y Gastric Bypass/Vertical Banded Gastroplasty	The Netherlands	168
59	Werling et al. 2012 [81]	Randomized Controlled Study	82	2	1	44.9	0.28	0.72	42.1	Roux-en-Y Gastric Bypass/Vertical Banded Gastroplasty	Sweden	120
60	Yale 1989 [82]	Prospective Cohort Study	537	9	5	36	0.162	0.838	46.8	Roux-en-Y GastroJejunostomy/Vertical Banded Gastroplasty/Gastrogastrotomy	USA	60
61	Zitsman et al. 2014 [83]	Prospective Cohort Study	137	2	1	17	0.31	0.69	48.3	Laproscopic Adjustable Gastric Banding	USA	60

**Table 2:** Meta-regression analysis on the sources of heterogeneity for the prevalence of mortality among bariatric surgery cohorts with reported suicide mortality.

Moderators	No. of Studies Used	Slope	Standard Error	Lower Limit (95% CI)	Upper Limit (95% CI)	*Z* Value	*p* Value
Mean Age	50	−0.02370	0.03244	−0.08729	0.03988	−0.73065	0.46499
Proportion of males	54	−0.01258	0.01069	−0.03353	0.00838	−1.17621	0.23951
Mean BMI	49	0.008284	0.03481	0.01461	0.15107	2.37980	0.01732 *
Longest follow-up interval	44	0.01177	0.00271	0.00646	0.01708	4.34545	0.00001 *

* *p* < 0.05 is considered significant. Mean BMI and Follow-up interval are significant moderators.

**Table 3:** Subgroup analysis on the sources of heterogeneity for the prevalence of mortality among bariatric surgery cohorts with reported suicide mortality.

Predictor	No. of Studies	Pooled Prevalence (%)	95% CI	*p*-Value in between Group Comparison
Restrictive procedures	25	1.4	0.9–2.3	0.309
Malabsorptive procedures	24	2.4	1.8–3.3	
Restrictive and/or Malabsorptive procedures	6	1.6	0.6–4.1	
Unspecified procedures	6	2.5	0.6–9.2	
Overall:	61	2.1	1.6–2.7	
Continent—North America	28	2.1	1.5–2.9	0.380
Continent—Europe	28	1.7	1.1–2.7	
Continent—South America	2	2.4	1.3–4.5	
Continent—Oceania	3	0.5	0.1–3.1	
Overall:	61	2.0	1.6–2.5	

**Table 4:** Subgroup analysis on the sources of heterogeneity for the prevalence of suicide.

Predictor	No. of Studies	Pooled Prevalence (%)	95% CI	*p*-Value in between Group Comparison
Restrictive procedures	25	0.5	0.3–0.8	0.131
Malabsorptive procedures	24	0.3	0.2–0.4	
Restrictive and/or Malabsorptive procedures	6	0.3	0.1–1.0	
Unspecified procedures	6	0.2	0.1–0.3	
Overall:	61	0.3	0.2–0.4	
Continent—North America	28	0.3	0.2–0.5	0.878
Continent—Europe	28	0.4	0.2–0.6	
Continent—South America	2	0.3	0.1–1.4	
Continent—Oceania	3	0.1	0–1.1	
Overall:	61	0.3	0.3–0.5	

## References

[1] World Health Organization (2017). Obesity. http://www.who.int/topics/obesity/en/.

[2] Sanjeev S., Raed H. (2017). Psychiatric Care in Severe Obesity: An Interdisciplinary Guide to Integrated Care.

[3] Rajan T.M., Menon V. (2017). Psychiatric disorders and obesity: A review of association studies. J. Postgrad. Med..

[4] (2017). Global Strategy on Diet, Physical Activity and Health. World Health Organization. http://www.who.int/dietphysicalactivity/en/.

[5] Senger E. (2011). Bariatric surgery guidelines in need of revision, experts argue. CMAJ.

[6] Maggard M.A., Shugarman L.R., Suttorp M., Maglione M., Sugerman H.J., Livingston E.H., Nguyen N.T., Li Z., Mojica W.A., Hilton L. (2005). Meta-analysis: Surgical treatment of obesity. Ann. Intern. Med..

[7] Angrisani L., Santonicola A., Iovino P., Formisano G., Buchwald H., Scopinaro N. (2015). Bariatric Surgery Worldwide 2013. Obes. Surg..

[8] Slomski A. (2017). Bariatric Surgery Has Durable Effects in Controlling Diabetes. JAMA.

[9] Tirado R., Masdeu M.J., Vigil L., Rigla M., Luna A., Rebasa P., Pareja R., Hurtado M., Caixàs A. (2017). Impact of Bariatric Surgery on Heme Oxygenase-1, Inflammation, and Insulin Resistance in Morbid Obesity with Obstructive Sleep Apnea. Obes. Surg..

[10] Neff K.J., Baud G., Raverdy V., Caiazzo R., Verkindt H., Noel C., le Roux C.W., Pattou F. (2017). Renal Function and Remission of Hypertension After Bariatric Surgery: A 5-Year Prospective Cohort Study. Obes. Surg..

[11] Cardoso L., Rodrigues D., Gomes L., Carrilho F. (2017). Short- and long-term mortality after bariatric surgery: A systematic review and meta-analysis. Diabetes Obes. Metab..

[12] Peterhänsel C., Petroff D., Klinitzke G., Kersting A., Wagner B. (2013). Risk of completed suicide after bariatric surgery: A systematic review. Obes. Rev..

[13] Tindle H.A., Omalu B., Courcoulas A., Marcus M., Hammers J., Kuller L.H. (2010). Risk of suicide after long-term follow-up from bariatric surgery. Am. J. Med..

[14] Hensel J., Selvadurai M., Anvari M., Taylor V. (2016). Mental Illness and psychotropic medication use among people assessed for bariatric surgery in Ontario, Canada. Obes. Surg..

[15] Yen Y.C., Huang C.K., Tai C.M. (2014). Psychiatric aspects of bariatric surgery. Curr. Opin. Psychiatry.

[16] Yusufov M., Dalrymple K., Bernstein M.H., Walsh E., Rosenstein L., Chelminski I., Zimmerman M. (2017). Body mass index, depression, and suicidality: The role of self-esteem in bariatric surgery candidates. J. Affect. Disord..

[17] Mitchell J.E., Crosby R., de Zwaan M., Engel S., Roerig J., Steffen K., Gordon K.H., Karr T., Lavender J., Wonderlich S. (2013). Possible risk factors for increased suicide following bariatric surgery. Obesity.

[18] Ho R.C., Ong H.S., Kudva K.G., Cheung M.W., Mak A. (2010). How to critically appraise and apply meta-analyses in clinical practice. Int. J. Rheum. Dis..

[19] Cheung M.W., Ho R.C., Lim Y., Mak A. (2012). Conducting a meta-analysis: Basics and good practices. Int. J. Rheum. Dis..

[20] Loh A.Z., Tan J.S., Zhang M.W., Ho R.C. (2017). The Global Prevalence of Anxiety and Depressive Symptoms among Caregivers of Stroke Survivors. J. Am. Med. Dir. Assoc..

[21] Ho R.C., Ong H., Thiaghu C., Lu Y., Ho C.S., Zhang M.W. (2016). Genetic Variants That Are Associated with Neuropsychiatric Systemic Lupus Erythematosus. J. Rheumatol..

[22] Lu Y., Andiappan A.K., Lee B., Ho R., Lim T.K., Kuan W.S., Goh D.Y.T., Mahadevan M., Sim T.B., Wang D.Y. (2016). Neuropeptide Y associated with asthma in young adults. Neuropeptides.

[23] Puthran R., Zhang M.W., Tam W.W., Ho R.C. (2016). Prevalence of depression amongst medical students: A meta-analysis. Med. Educ..

[24] Aarts E.O., Dogan K., Koehestanie P., Aufenacker T.J., Janssen I.M., Berends F.J. (2014). Long-term results after laparoscopic adjustable gastric banding: A mean fourteen year follow-up study. Surg. Obes. Relat. Dis..

[25] Adams T.D., Gress R.E., Smith S.C., Halverson R.C., Simper S.C., Rosamond W.D., LaMonte M.J., Stroup A.M., Hunt S.C. (2007). Long-term mortality after gastric bypass surgery. N. Engl. J. Med..

[26] Adams T.D., Davidson L.E., Litwin S.E., Kolotkin R.L., LaMonte M.J., Pendleton R.C., Strong M.B., Vinik R., Wanner N.A., Hopkins P.N. (2012). Health benefits of gastric bypass surgery after 6 years. JAMA.

[27] Arapis K., Chosidow D., Lehmann M., Bado A., Polanco M., Kamoun-Zana S., Pelletier A.L., Kousouri M., Marmuse J.-P. (2012). Long-term results of adjustable gastric banding in a cohort of 186 super-obese patients with a BMI ≥ 50 kg/m^2^. J. Visc. Surg..

[28] Arman G.A., Himpens J., Dhaenens J., Ballet T., Vilallonga R., Leman G. (2016). Long-term (11+years) outcomes in weight, patient satisfaction, comorbidities, and gastroesophageal reflux treatment after laparoscopic sleeve gastrectomy. Surg. Obes. Relat. Dis..

[29] Biertho L., Biron S., Hould F.S., Lebel S., Marceau S., Marceau P. (2010). Is biliopancreatic diversion with duodenal switch indicated for patients with body mass index <50 kg/m^2^?. Surg. Obes. Relat. Dis..

[30] Bolckmans R., Himpens J. (2016). Long-term (>10 years) Outcome of the Laparoscopic Biliopancreatic Diversion with Duodenal Switch. Ann. Surg..

[31] Busetto L., Mirabelli D., Petroni M.L., Mazza M., Favretti F., Segato G., Chiusolo M., Merletti F., Balzola F., Enzi G. (2007). Comparative long-term mortality after laparoscopic adjustable gastric banding versus nonsurgical controls. Surg. Obes. Relat. Dis..

[32] Busetto L., De Stefano F., Pigozzo S., Segato G., De Luca F., Favretti F. (2014). Long-term cardiovascular risk and coronary events in morbidly obese patients treated with laparoscopic gastric banding. Surg. Obes. Relat. Dis..

[33] Cadière G.-B., Himpens J., Bazi M., Cadière B., Vouche M., Capelluto E., Dapri G. (2011). Are laparoscopic gastric bypass after gastroplasty and primary laparoscopic gastric bypass similar in terms of results?. Obes. Surg..

[34] Capella J.F., Capella R.F. (1996). The weight reduction operation of choice: Vertical banded gastroplasty or gastric bypass?. Am. J. Surg..

[35] Carelli A.M., Youn H.A., Kurian M.S., Ren C.J., Fielding G.A. (2010). Safety of the laparoscopic adjustable gastric band: 7-year data from a U.S. center of excellence. Surg. Endosc..

[36] Christou N.V., Look D., Maclean L.D. (2006). Weight gain after short- and long-limb gastric bypass in patients followed for longer than 10 years. Ann. Surg..

[37] Cobourn C., Chapman M.A., Ali A., Amrhein J. (2013). Five-year weight loss experience of outpatients receiving laparoscopic adjustable gastric band surgery. Obes. Surg..

[38] Diniz Mde F., Moura L.D., Kelles S.M., Diniz M.T. (2013). Long-term mortality of patients submitted to Roux-en-Y gastric bypass in Public Health System: High prevalence of alcoholic cirrhosis and suicides. Arq. Bras. Cir. Dig..

[39] Goldfeder L.B., Ren C.J., Gill J.R. (2006). Fatal complications of bariatric surgery. Obes. Surg..

[40] Gribsholt S.B., Thomsen R.W., Svensson E., Richelsen B. (2017). Overall and cause-specific mortality after Roux-en-Y gastric bypass surgery: A nationwide cohort study. Surg. Obes. Relat. Dis..

[41] Higa K.D., Boone K.B., Ho T. (2000). Complications of the laparoscopic Roux-en-Y gastric bypass: 1040 patients—What have we learned?. Obes. Surg..

[42] Himpens J., Cadière G.B., Bazi M., Vouche M., Cadière B., Dapri G. (2011). Long-term outcomes of laparoscopic adjustable gastric banding. Arch. Surg..

[43] Himpens J., Verbrugghe A., Cadière G.B., Everaerts W., Greve J.W. (2012). Long-term results of laparoscopic Roux-en-Y Gastric bypass: Evaluation after 9 years. Obes. Surg..

[44] Bruschi Kelles S.M., Diniz M.F., Machado C.J., Barreto S.M. (2014). Mortality rate after open Roux-in-Y gastric bypass: A 10-year follow-up. Braz. J. Med. Biol. Res..

[45] Kral J.G., Görtz L., Hermansson G., Wallin G.S. (1993). Gastroplasty for obesity: Long-term weight loss improved by vagotomy. World J. Surg..

[46] Lagerros Y.T., Brandt L., Hedberg J., Sundbom M., Bodén R. (2017). Suicide, Self-harm, and Depression after Gastric Bypass Surgery: A Nationwide Cohort Study. Ann. Surg..

[47] Lemanu D.P., Singh P.P., Rahman H., Hill A.G., Babor R., MacCormick A.D. (2015). Five-year results after laparoscopic sleeve gastrectomy: A prospective study. Surg. Obes. Relat. Dis..

[48] MacDonald K.G., Long S.D., Swanson M.S., Brown B.M., Morris P., Dohm G.L., Pories W.J. (1997). The gastric bypass operation reduces the progression and mortality of non-insulin-dependent diabetes mellitus. J. Gastrointest. Surg..

[49] MacLean L.D., Rhode B.M., Nohr C.W. (2000). Late outcome of isolated gastric bypass. Ann. Surg..

[50] Marceau P., Biron S., Hould F.-S., Lebel S., Marceau S., Lescelleur O., Biertho L., Simard S. (2007). Duodenal switch: Long-term results. Obes. Surg..

[51] Marceau P., Biron S., Hould F.-S., Lebel S., Marceau S., Lescelleur O., Biertho L., Simard S. (2009). Duodenal switch improved standard biliopancreatic diversion: A retrospective study. Surg. Obes. Relat. Dis..

[52] Marceau P., Biron S., Marceau S., Hould F.S., Lebel S., Lescelleur O., Biertho L., Simard S., Kral J.G. (2015). Long-Term Metabolic Outcomes 5 to 20 Years After Biliopancreatic Diversion. Obes. Surg..

[53] Marsk R., Näslund E., Freedman J., Tynelius P., Rasmussen F. (2010). Bariatric surgery reduces mortality in Swedish men. Br. J. Surg..

[54] McPhee J., Khlyavich Freidl E., Eicher J., Zitsman J.L., Devlin M.J., Hildebrandt T., Sysko R. (2015). Suicidal Ideation and Behaviours Among Adolescents Receiving Bariatric Surgery: A Case-Control Study. Eur. Eat. Disord. Rev..

[55] Mitchell J.E., Lancaster K.L., Burgard M.A., Howell L.M., Krahn D.D., Crosby R.D., Wonderlich S.A., Gosnell B.A. (2001). Long-term follow-up of patients’ status after gastric bypass. Obes. Surg..

[56] Näslund E., Granström L., Stockeld D., Backman L. (1994). Marlex Mesh Gastric Banding: A 7–12 Year Follow-up. Obes. Surg..

[57] Näslund E., Backman L., Granström L., Stockeld D. (1995). Does the Size of the Upper Pouch Affect Weight Loss after Vertical Banded Gastroplasty. Obes. Surg..

[58] Nocca D., Krawczykowsky D., Bomans B., Noël P., Picot M.C., Blanc P.M., de Seguin de Hons C., Millat B., Gagner M., Monnier L. (2008). A prospective multicenter study of 163 sleeve gastrectomies: Results at 1 and 2 years. Obes. Surg..

[59] Obeid N.R., Malick W., Concors S.J., Fielding G.A., Kurian M.S., Ren-Fielding C.J. (2016). Long-term outcomes after Roux-en-Y gastric bypass: 10- to 13-year data. Surg. Obes. Relat. Dis..

[60] O’Brien P.E., MacDonald L., Anderson M., Brennan L., Brown W.A. (2013). Long-term outcomes after bariatric surgery: Fifteen-year follow-up of adjustable gastric banding and a systematic review of the bariatric surgical literature. Ann. Surg..

[61] Omalu B.I., Ives D.G., Buhari A.M., Lindner J.L., Schauer P.R., Wecht C.H., Kuller L.H. (2007). Death rates and causes of death after bariatric surgery for Pennsylvania residents, 1995 to 2004. Arch. Surg..

[62] Peeters A., O’Brien P.E., Laurie C., Anderson M., Wolfe R., Flum D., MacInnis R.J., English D.R., Dixon J. (2007). Substantial intentional weight loss and mortality in the severely obese. Ann. Surg..

[63] Pekkarinen T., Koskela K., Huikuri K., Mustajoki P. (1994). Long-term Results of Gastroplasty for Morbid Obesity: Binge-Eating as a Predictor of Poor Outcome. Obes. Surg..

[64] Pories W.J., MacDonald K.G., Flickinger E.G., Dohm G.L., Sinha M.K., Barakat H.A., May H.J., Khazanie P., Swanson M.S., Morgan E. (1992). Is type II diabetes mellitus (NIDDM) a surgical disease?. Ann. Surg..

[65] Pories W.J., Swanson M.S., MacDonald K.G., Long S.B., Morris P.G., Brown B.M., Barakat H.A., de Ramon R.A., Israel G., Dolezal J.M. (1995). Who would have thought it? An operation proves to be the most effective therapy for adult-onset diabetes mellitus. Ann. Surg..

[66] Powers P.S., Boyd F., Blair C.R., Stevens B., Rosemurgy A. (1992). Psychiatric Issues in Bariatric Surgery. Obes. Surg..

[67] Powers P.S., Rosemurgy A., Boyd F., Perez A. (1997). Outcome of gastric restriction procedures: Weight, psychiatric diagnoses, and satisfaction. Obes. Surg..

[68] Rawlins L., Rawlins M.P., Brown C.C., Schumacher D.L. (2013). Sleeve gastrectomy: 5-year outcomes of a single institution. Surg. Obes. Relat. Dis..

[69] Van Rutte P.W., Smulders J.F., de Zoete J.P., Nienhuijs S.W. (2014). Outcome of sleeve gastrectomy as a primary bariatric procedure. Br. J. Surg..

[70] Shah K., Johnny Nergard B., Stray Frazier K., Geir Leifsson B., Aghajani E., Gislason H. (2016). Long-term effects of laparoscopic Roux-en-Y gastric bypass on metabolic syndrome in patients with morbid obesity. Surg. Obes. Relat. Dis..

[71] Sieber P., Gass M., Kern B., Peters T., Slawik M., Peterli R. (2014). Five-year results of laparoscopic sleeve gastrectomy. Surg. Obes. Relat. Dis..

[72] Skroubis G., Karamanakos S., Sakellaropoulos G., Panagopoulos K., Kalfarentzos F. (2011). Comparison of early and late complications after various bariatric procedures: Incidence and treatment during 15 years at a single institution. World J. Surg..

[73] Smith S.C., Goodman G.N., Edwards C.B. (1995). Roux-en-Y Gastric Bypass: A 7-year Retrospective Review of 3,855 Patients. Obes. Surg..

[74] Smith S.C., Edwards C.B., Goodman G.N., Halversen R.C., Simper S.C. (2004). Open vs laparoscopic Roux-en-Y gastric bypass: Comparison of operative morbidity and mortality. Obes. Surg..

[75] Suter M., Calmes J.M., Paroz A., Giusti V. (2006). A 10-year experience with laparoscopic gastric banding for morbid obesity: High long-term complication and failure rates. Obes. Surg..

[76] Suter M., Donadini A., Romy S., Demartines N., Giusti V. (2011). Laparoscopic Roux-en-Y gastric bypass: Significant long-term weight loss, improvement of obesity-related comorbidities and quality of life. Ann. Surg..

[77] Svenheden K.E., Akesson L.A., Holmdahl C., Näslund I. (1997). Staple disruption in vertical banded gastroplasty. Obes. Surg..

[78] Tao W., Plecka-Östlund M., Lu Y., Mattsson F., Lagergren J. (2015). Causes and risk factors for mortality within 1 year after obesity surgery in a population-based cohort study. Surg. Obes. Relat. Dis..

[79] Thereaux J., Corigliano N., Poitou C., Oppert J.M., Czernichow S., Bouillot J.L. (2015). Five-year weight loss in primary gastric bypass and revisional gastric bypass for failed adjustable gastric banding: Results of a case-matched study. Surg. Obes. Relat. Dis..

[80] Van de Weijgert E.J., Ruseler C.H., Elte J.W. (1999). Long-term follow-up after gastric surgery for morbid obesity: Preoperative weight loss improves the long-term control of morbid obesity after vertical banded gastroplasty. Obes. Surg..

[81] Werling M., Fändriks L., Björklund P., Maleckas A., Brandberg J., Lönroth H., Le Roux C.W., Olbers T. (2013). Long-term results of a randomized clinical trial comparing Roux-en-Y gastric bypass with vertical banded gastroplasty. Br. J. Surg..

[82] Yale C.E. (1989). Gastric surgery for morbid obesity. Complications and long-term weight control. Arch. Surg..

[83] Zitsman J.L., DiGiorgi M.F., Fennoy I., Kopchinski J.S., Sysko R., Devlin M.J. (2015). Adolescent laparoscopic adjustable gastric banding (LAGB): Prospective results in 137 patients followed for 3 years. Surg. Obes. Relat. Dis..

[84] Chang S.H., Stoll C.R., Song J., Varela J.E., Eagon C.J., Colditz G.A. (2013). The effectiveness and risks of bariatric surgery: An updated systematic review and meta-analysis, 2003–2012. JAMA Surg..

[85] Rottenstreich A., Keidar A., Yuval J.B., Abu-Gazala M., Khalaileh A., Elazary R. (2016). Outcome of bariatric surgery in patients with type 1 diabetes mellitus: Our experience and reviewof the literature. Surg. Endosc..

[86] Balla A., Batista Rodríguez G., Corradetti S., Balagué C., Fernández-Ananín S., Targarona E.M. (2017). Outcomes after bariatric surgery according to large databases: A systematic review. Langenbecks Arch. Surg..

[87] World Health Organization http://www.who.int/mental_health/prevention/suicide/suicideprevent/en/.

[88] Adams T.D., Mehta T.S., Davidson L.E., Hunt S.C. (2015). All-Cause and Cause-Specific Mortality Associated with Bariatric Surgery: A Review. Curr. Atheroscler. Rep..

[89] Roizblatt A., Roizblatt D., Soto-Aguilar B.F. (2016). Suicide risk after bariatric surgery. Rev. Med. Chile.

[90] Greenberg I., Sogg S., Perna F.M. (2009). Behavioral and psychological care in weight loss surgery: Best practice update. Obesity.

[91] Padwal R.S., Klarenbach S.W., Wang X., Sharma A.M., Karmali S., Birch D.W., Majumdar S.R. (2013). A simple prediction rule for all-cause mortality in a cohort eligible for bariatric surgery. JAMA Surg..

